# The prevalence of menstrual disorders and premenstrual syndrome among adolescent girls living in North Borneo, Malaysia: a questionnaire-based study

**DOI:** 10.1186/s12905-022-01929-1

**Published:** 2022-08-13

**Authors:** Jerilee Mariam Khong Azhary, Lai Kim Leng, Nuguelis Razali, Sofiah Sulaiman, Ana Vetriana Abd Wahab, Aizura Syafinaz Ahmad Adlan, Jamiyah Hassan

**Affiliations:** 1 Department of Obstetrics and Gynaecology, Faculty of Medicine, University of Malaya, 50603 Kuala Lumpur, Malaysia; 2 Department of Obstetrics and Gynaecology, Sabah Women and Children Hospital, 88996 Kota Kinabalu, Sabah Malaysia

**Keywords:** Adolescents, Dysmenorrhoea, Premenstrual syndrome, Reproductive health, Rural population

## Abstract

**Background:**

This study aimed to determine menstrual characteristics and related morbidities among adolescent girls living in Sabah, North Borneo, a less-developed state in Malaysia.

**Methods:**

Data were obtained from a quantitative survey conducted in three government high schools located in Ranau, Sabah. The participants were adolescent girls who had attained menarche between the ages of 14 and 19.

**Results:**

Based on the analysis of questionnaires completed by 757 adolescent girls, the mean age at the time of the survey was 17 ± 1.4 years, and the mean menarche age was 12.2 ± 1.1 years. Our data demonstrated that 85.7% of the participants experienced dysmenorrhoea, of which at least 42.1% (mean pain score ± SD: 4.81 ± 0.76, 95% confidence interval (CI) 4.72, 4.90) experienced moderate dysmenorrhoea, and 11.2% (mean pain score ± SD: 7.86 ± 0.94, 95% CI 7.64, 8.08) experienced severe dysmenorrhoea. Over 70% of these girls complained of tiredness, headache, and appetite changes during menses.

**Conclusions:**

The prevalence of menstrual disorders and related morbidities was high among the girls residing in Sabah. Reproductive health issues in rural and socioeconomically deprived areas remains poorly addressed. The main consequence of neglecting menstrual disorders and their related morbidities is impaired future sexual reproductive health in adults. Thus, addressing adolescent reproductive health issues is crucial, especially for girls living in areas where access to healthcare is difficult. The information gathered from this study can be used to strategize effective interventions to improve adolescents' reproductive health status in rural areas.

## Background

According to the World Health Organization (WHO), adolescents are individuals aged between 10 and 19 years [1]. Secondary sexual characteristics, sexual maturation, and reproductive capacity are usually attained in this age range. In girls, ovulation and menstruation begin during this period [2–5] and are often accompanied by menstrual disorders and related morbidities [4–6].

Inequality in a country’s socioeconomic status can lead to progressive health issues [7]. In Malaysia, lower socioeconomic status is often more prevalent in rural areas [8]. The socioeconomic status of a population influences the prevalence of menstrual disorders and their associated morbidities [9–11]. Malaysia provides a unique setting for studying menstrual cycle characteristics. Malaysia is divided into two halves: the South China Sea peninsula and North Borneo states (Sabah and Sarawak). The gap in socioeconomic status between these two regions is huge [12, 13]. The peninsula is more populated and more socioeconomically developed than Sabah and Sarawak [13]. The poverty rates in Sabah and Sarawak were 8.1% and 2.4% in 2012, respectively, higher than most states in the peninsula [14]. Although Malaysia is relatively free from natural disasters, Sabah, the most impoverished state in Malaysia, was affected by a series of earthquakes in 2015, which further complicated its socioeconomic status.

Adolescent girls from urban areas and higher socioeconomic populations have a higher prevalence of menstrual disorders such as premenstrual syndrome and dysmenorrhoea [15–19]. Girls with menstrual disorders frequently report other concurrent somatic symptoms such as headaches, fatigue, and vomiting [20, 21]. This inevitably leads to increased over-the-counter drug usage, school absenteeism, and a poorer quality of life. Data retrieved from the Peninsula of Malaysia revealed that the prevalence of premenstrual syndrome and dysmenorrhoea exceeded 60% and 70%, respectively [15, 20, 23–28]. Compared to the rural states in the peninsula, girls from the metropolitan city of Kuala Lumpur had a higher prevalence of dysmenorrhoea and increased school absenteeism [20]. These findings are attributed to the fact that girls from rural areas are more accepting of their condition and more tolerant of pain than girls from urban areas [15, 16].

To date, no study has specifically evaluated menstrual patterns and related morbidities in girls living in Sabah. This study aimed to determine the menstrual characteristics and associated symptoms experienced by adolescent girls residing in Ranau, Sabah, a state below the poverty line and recently hit by multiple earthquakes. Achieving wholesome reproductive health in adolescents is crucial for ensuring a healthy reproductive system in adult women [27]. Therefore, addressing the menstrual challenges faced by rural girls should be prioritised, because healthcare facilities and their related resources may not be easily accessible.

## Methods

### Subjects and settings

This questionnaire-based study was conducted in Ranau, a district on the west coast of Sabah in North Borneo, Malaysia. After obtaining approval from the Department of Education in Sabah and Malaysia's Ministry of Education, three schools were randomly selected using computer-generated randomisation. The sample size for this study was calculated using the Kish formula for prevalence objectives [29]. With an assumption of 75% prevalence of menstrual disorders among adolescents, the required sample size was approximately 750 [24, 25, 28]. We met with school principals to explain the purpose of this study. The names of each school and student were kept confidential by replacing them with their serial numbers. Student participation was voluntary, and the research activities did not disrupt the school activities or teaching sessions. Three schools agreed to participate and female students who had attained menarche were approached. Two research trips were conducted between January 4^th^ and February 2^nd^, 2016. The schoolgirls were given an information sheet and a parental consent form. Those who returned the parental consent form were recruited and assembled in the school hall during two visits. The principal investigator provided a brief explanation of the study and described the contents of the questionnaire. When completing the questionnaires in the hall, the participants were encouraged to clarify any doubts pertaining to the questions by asking one of the six gynaecologically-trained, on-site research assistants. All completed questionnaires were collected from students before leaving the hall to obtain the highest possible response rate.

### Questionnaire

The questionnaires used in this study were adapted from Parker et al. [30] and were prepared in two languages: English and Malay. The questionnaires were composed of five sections: Sect. 1 gathered general information, including date of birth, age at menarche, height, and weight. Sections 2 and 3 collected data regarding menstrual patterns and somatic symptoms related to menses, respectively. Section 4 collected the students' perceptions towards their menses, and the final section identified allergies and intolerance to any food during menses. The validated English version of the questionnaire was translated into the Malay language. The Malay version of the questionnaire was verified via back-translation by an expert from the Faculty of Language and Linguistics at the University of Malaya. It was pre-tested with 20 students prior to the actual study.

#### Ethical oversight

Ethical insights were provided by the University of Malaya Medical Research Ethical Committee (MREC no. 20157-1601) on September 7th, 2015.

#### Statistical analysis

The data were analysed using SPSS Statistics for Windows, version 17.0 (SPSS Inc., Chicago, Ill., USA). The mean value was used for normally distributed continuous variables. The standard deviation (SD), a summary measure of the differences between each observation and the mean, was also calculated. The 95% confidence interval (CI) was calculated for each independent variable.

## Results

At the time of the study, 1814 female students attended the three participant schools in the Ranau District of Sabah. A total of 781 questionnaires were distributed to students who volunteered to participate. Of the distributed questionnaires, 24 were incomplete or were blank. Therefore, this cross-sectional study gathered data from 757 students with a response rate of 96.9%. The mean age of the participants was 17 ± 1.4 years, whereas 12.2 ± 1.1 years was the mean age of menarche. Demographic characteristics of the participants are presented in Table 1.

**Table 1:** Demographic data

Age (mean SD)	17 ± 1.4
*Age breakdown, n (%)*	
14–15	110 (14.6)
16–17	515 (68.2)
18–19	131 (17.2)
Weight (mean ± SD)	49 ± 8
Height (mean ± SD)	154 ± 6
*BMI breakdown, n (%)*	
Underweight	22 (3.1)
Normal weight	631 (89.5)
Overweight-Obese	52 (7.4)
Age of menarche (mean SD) 12.2 ± 1.1	
*Age of menarche breakdown, n (%)*	
9–10	33 (4.5)
11–12	438 (59.2)
13–14	260 (35.1)
15–16	9 (1.2)
Above 17	1 (0.1)

### Typical menstrual bleeding characteristics

**Fig. 1:**
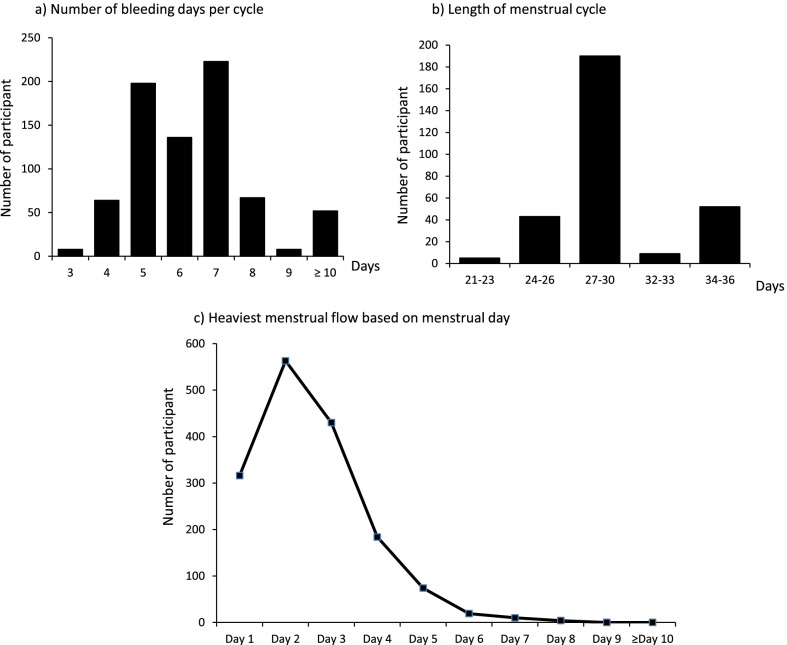
Typical menstrual characteristics: **a** Number of bleeding days per cycle, **b** Length of menstrual cycle and **c** heaviest menstrual flow based on menstrual day

Figure 1 illustrates the number of bleeding days in each cycle (Fig. 1a), cycle length (Fig. 1b), and heaviest menstrual flow based on the menstrual day (Fig. 1c). The majority of participants experienced a normal number of bleeding days per cycle, with a mean of 6.32 ± 1.56 days. Approximately 7.0% (n = 53/757) of the students experienced bleeding for ≥ 10 days (Fig. 1a). 39.4% (n = 299/757) of students were able to report their menstrual cycle length. From this number, 190 students reported having a regular menstrual cycle of between 27 and 30 days. Meanwhile, 61 students reported that their menstrual cycles exceeded 30 days, as illustrated in Fig. 1b. The heaviest menstrual bleeding was reported between day two and three of their menses (Fig. 1c).

### Dysmenorrhoea

**Fig. 2:**
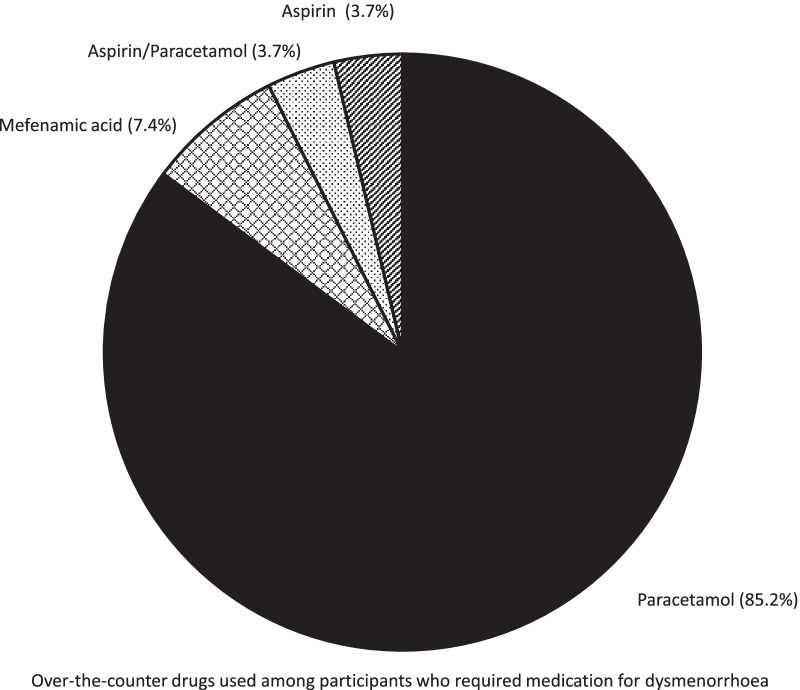
Over-the-counter drugs used among participants who required medication for dysmenorrhoea

Dysmenorrhoea was reported by 85.7% (n = 648/756) of students, causing 16.9% (n = 110/648) to skip school. Severity of dysmenorrhoea was categorised according to a rating scale from 0 to 10; no/mild pain (0–3), moderate (4–7), and severe (8–10). Seventy-three students (11.2%) experienced severe dysmenorrhoea with a mean pain score of 7.86 ± 0.94 (95% CI 7.64, 8.08). Meanwhile, 273 students stated that they experienced moderate dysmenorrhoea with a mean pain score of 4.81 ± 0.76 (95% CI 4.72, 4.90), as illustrated in Table 2. However, only 8.3% (n = 54/648) of students who experienced menstrual-related pain consumed oral analgesia or regular hormonal pills to control pain. Among those who required analgesia, the preferred choices were paracetamol (85.2%) and nonsteroidal anti-inflammatory drugs (7.4%) (Fig. 2). Apart from pain, the top three reasons for not attending school were heavy menstrual bleeding (0.03%), nausea, and vomiting (0.01%).

**Table 2:** Prevalence and the severity of dysmenorrhea

Prevalence of dysmenorrhea, n = 756		
No, n (%)	108	14.3
Yes, n (%)	648	85.7

Severity of dysmenorrhea breakdown, n = 648					
	n (%)	Mean	SD	95%CI	
Severe	73 (11.2)	7.86	0.94	7.64	8.08
Moderate	273 (42.1)	4.81	0.76	4.72	4.90
Mild	302 (46.6)	2.00	0.79	1.91	2.09

### Menstrual-related symptoms

The top ten symptoms related to menstruation documented by the students were tiredness, change in appetite, headache, depression, frequent urination, bloating, pelvic pain, farting pain, vomiting, and anal bleeding, as described in Table 3. In particular, 82.5% (n = 623/755) of the girls complained of tiredness during menses, 75.7% (n = 569/751) observed a change in their appetite, and 69.2% (n = 522/754) experienced headaches during their bleeding days. Over half of the participants reported feeling depressed and/or experiencing increased urination (Table 3).

**Table 3:** Top ten symptoms reported

Symptoms	Frequency, n (%)	Number of respondents, n	Missing data, n	95% CI
Tiredness	623 (82.5)	755	2	0.80, 0.85
Change of appetite	569 (75.7)	751	6	0.73, 0.71
Headache	522 (69.2)	754	3	0.66, 0.71
Depression	426 (56.4)	755	2	0.53, 0.58
Frequent urination	412 (54.7)	752	5	0.52, 0.57
Bloating	198 (26.5)	747	10	0.23, 0.28
Pelvic pain	178 (24.1)	719	38	0.22, 0.26
Farting pain	101 (13.9)	726	31	0.12, 0.15
Vomiting	53 (7.0)	755	2	0.05, 0.07
Anal bleeding	47 (6.3)	747	10	0.04, 0.07

### Diagnosis and discussion of dysmenorrhoea

Regarding the diagnosis and investigation of dysmenorrhoea, 62 (8.2%) students consulted their general practitioner, 15 (1.9%) girls visited a gynaecologist, and 25 (3.3%) sought aid from traditional healers in their villages. Eighteen students reported menstrual pain. Blood tests and ultrasonography were performed in nine and three students, respectively, and six students had undergone surgery for pain. Additionally, majority of the students discussed menstrual issues with their friends (72.5%, n = 549/757) and family (60.8%, n = 460/757), instead of consulting their teachers or counsellors (4.6%, n = 35/756).

## Discussion

Our data showed a dysmenorrhoea prevalence of 85.7% among our participants, with more than half reporting moderate-to-severe pain. Additionally, menstruation-related somatic symptoms, such as tiredness, changes in appetite, and headache, were common complaints.

The prevalence of dysmenorrhoea among adolescents worldwide varies between 16 and 93% [31, 32] and is greatly influenced by geographic location. For example, the prevalence of dysmenorrhoea is 94% in Oman, 59.8% in Bangladesh, 34% in Egypt, and 0.9% in Korea [32]. In the present study, 85.7% of participants experienced dysmenorrhoea. The study participants had a higher prevalence of dysmenorrhoea than their counterparts living in the Malaysian Peninsula. For example, the prevalence in Negeri Sembilan was 67.7% [23], 76.6% in Kelantan [24], and between 63.9 and 74.5% in the metropolitan city of Kuala Lumpur [25, 28] (Table 4).

**Table 4:** Previously reported of dysmenorrhoea in adolescents living in the peninsula of Malaysia

Authors	Year of publication	Location of study	Poverty rate in study area (%) (approximately)	Subject age (mean ± SD and/or range)	Sample size	Prevalence of dysmenorrhoea (%)
Wong et al. [20]	2010	Kuala Lumpur	1.1	15.19 ± 1.39 (13–19)	1092	46.9
Lee et al. [23]	2003	7 distrcits in Negeri sembilan	0.7	15.4 (± 1.8)	2411	67.7
Wong et al. [24]	2011	2 districts in Kelantan	2.7	15.28 ± 1.45 (13–19)	1295	76.1
Wong et al. [25]	2009	Kuala Lumpur	1.1	15.19 ± 1.39	1075	74.5
Liliwati et al. [26]	2007	Hulu Langat, Selangor	0.8	12–17	300	62.3
Mariappen y [28]	2022	Kuala Lumpur	1.1	12.14 ± 1.11	729	63.9

The findings reported in this study contradict the notion highlighted in other studies that rural girls suffer fewer menstrual disorders than urban girls [16–18, 20]. There are several possible explanations for this discrepancy in the results. First, the studies conducted on the Peninsula of Malaysia involved younger adolescents (mean age 15 years), whereas the mean age of the participants in the present study was 17 years. Interestingly, the prevalence of dysmenorrhoea increases with increasing gynaecological age (age after menarche) [33, 34]. Valvaikar et al. similarly reported that rural girls exhibited a higher prevalence of dysmenorrhoea than urban girls [35]. They added that the rationale behind this phenomenon was the rapid progress and urbanisation of rural areas, leading to a higher consumption of westernised high-fat foods combined with a sedentary lifestyle [35]. Third, some variations could exist in the methodology, definition of dysmenorrhoea, and study population adopted between these studies [19].

Additionally, menstrual disorders such as dysmenorrhoea are strongly associated with calamity-induced post-traumatic stress disorder (PTSD) [36]. Ranau was hit by a series of earthquakes that occurred less than a year before this study. The interconnection between PTSD and dysmenorrhoea is not fully understood. However, PTSD has been associated with low-grade systemic inflammation, as evidenced by increased levels of circulating cytokines, tumour necrosis factor, and interleukin-1 [36, 37]. The production of pain mediators, such as prostaglandin E2, can be induced by these proinflammatory cytokines [38]. Despite being in a country that is relatively free from such natural disasters, adolescents residing in Sabah suffer from a significant adverse effect on life satisfaction because of PTSD [39].

Although girls living in Sabah experienced a higher prevalence of dysmenorrhoea, they were less likely to skip school due to pain or to consume oral analgesia than girls living in the Malaysian Peninsula. More than 50% of young girls in Kuala Lumpur received oral analgesia to relieve menstrual pain [20], whereas only 8.3% of young girls in Sabah consumed oral analgesia. In addition to dysmenorrhoea, the prevalence of somatic symptoms during menstruation was equally high among our study participants. In the present study, 82.5% of participants felt tired, suffered from headaches (69.2%), and noted a change in appetite (75.7%) during menses. In contrast, the prevalence of such somatic symptoms, particularly tiredness and headache, among girls residing in Kuala Lumpur was lower (75.4% and 38.4%, respectively) [20]. Additionally, our study also noted that the girls living in Sabah were also less likely to utilised health care services with only 10.1% of these girls receiving healthcare consultation or intervention.

There are several explanations for these health-seeking behaviours. First, the vast differences in pain self-management could be attributed to girls from rural areas being more resilient and having higher endurance of menstrual disorders [15, 16]. Additionally, these girls may perceive menstrual pain as a normal part of their menstrual cycle [24]. Adolescent girls may not be aware of the existence of dedicated adolescent healthcare services available to them [24, 40]. Those who were aware of such health care services described embarrassment and lack of confidentiality as some of the major barriers to the utilisation of health care services for their sexual reproductive health [39–41]. Many find it difficult or uncomfortable to discuss menstruation-related issues, suggesting that sexually related topics are sensitive to Malaysian culture [40].

Second, due to their remoteness and diversity in language, ethnicity, and cultural background, people in Sabah face healthcare challenges [39, 42] including poor access to healthcare [43] and poor awareness of many non-communicable and communicable diseases [44]. A recent study has demonstrated that the utilisation of healthcare services in rural areas of Sabah was 48% as compared to 67.7% in peninsular Malaysia, which is attributed to the longer travel time and distance to assess healthcare [45]. When coupled with the lack of knowledge and awareness of health problems related to menstruation among these girls, they experience further reduction in the utilisation of health care services [40, 46]. Finally, the Ministry of Health, Malaysia has reported that current adolescent health services in Malaysia are not adolescent-friendly [47] and are therefore underutilized [40, 47]. Negative attitudes of healthcare providers and lack of privacy [41] were identified as some of the major barriers to the utilisation of adolescent health services. When dealing with adolescents, highly trained, knowledgeable, and competent professionals are significantly better at relating to adolescents. Engaging with adolescents in managing their health problems requires training of various levels of healthcare personnel [47]. Unfortunately, only 4.62% of the Ministry of Health’s budget was dedicated to healthcare personnel training programs in adolescent health [48].

During a focus group discussion among girls residing in rural and urban areas across the Malaysian Peninsula, urban girls commonly cited their mothers, sisters, peers, and teachers as sources of information about menstruation-related matters [15]. However, the participants preferred discussing menstrual-related matters with their peers. Similarly, those from rural areas admitted feeling embarrassed to discuss menstrual-related issues with their mothers and sisters, and thus prefer to talk about it with a friend [20]. A similar outcome was observed in the present study. It is clear that between sophomore (15–16 years old) to senior year (17–18 years old) communication and support transitions from mothers to friends [49].

To the best of our knowledge, this study is the first to describe menstrual characteristics and related morbidities in adolescent girls living in North Borneo, Malaysia. This study provides evidence that there is an urgent need to effectively address and develop interventions to improve the reproductive-related health status among rural girls in Sabah.

However, the results of this study should be interpreted with caution because of the small number of participants. Therefore, it does not represent the entire state of Sabah, which has 219 government high schools covering an area of 73,631 km^2^. Additionally, in this study, primary and secondary dysmenorrhoea and participants' socioeconomic status were not differentiated or recorded.

Despite the governments' best efforts, healthcare facilities, healthcare provider training, and other associated resources are still lacking [42, 43]. WHO recommends improving the quality of health service provision to adolescents by developing an adolescent-friendly national quality standard of care [50]. However, implementing such programs requires a deeper understanding of the issue [51]. This study provides insights into reproductive health challenges faced by adolescent girls in Sabah.

## Conclusion

In conclusion, girls residing in Sabah reported a higher prevalence of dysmenorrhoea with several menstruation-related somatic symptoms than their peninsular counterparts. Girls living in the Peninsula of Malaysia have better accessibility to essential sexual reproductive health information and specialist healthcare, and girls from Sabah lack these privileges. Information gathered from this study is required to strategize an effective intervention to improve adolescents' reproductive health status in rural areas, where reproductive health care, education, and essential information are sparse.

## Data Availability

The datasets used and analysed during the current study are available from the corresponding author upon reasonable request.
